# Impact of maternal body mass index on outcomes of singleton pregnancies after assisted reproductive technology: a 14-year analysis of the US Nationwide Inpatient Sample

**DOI:** 10.1186/s12884-023-05620-7

**Published:** 2023-04-26

**Authors:** Yi-Ping Li, Wei-Jiun Li, Wen-Chi Hsieh, Li-Shan Chen, Cheng-Wei Yu

**Affiliations:** 1 Department of Obstetrics and Gynecology, Shin Kong Wu Ho-Su Memorial Hospital, No. 95 Wen Chang Road, Shih-Lin District, Taipei, 111 Taiwan; 2 Department of Obstetrics and Gynecology, National Taiwan University Hospital and National Taiwan University College of Medicine, Taipei, Taiwan; 3 School of Medicine, Fu-Jen Catholic University, New Taipei City, Taiwan

**Keywords:** Assisted reproductive technology (ART), Body mass index (BMI), Nationwide Inpatient Sample (NIS), Obesity, Pregnancy

## Abstract

**Background:**

Obesity is increasing globally, which affects multiple human functions, including reproductive health. Many women with overweight and obesity of child-bearing years are treated with assisted reproductive technology (ART). However, the clinical impact of body mass index (BMI) on pregnancy outcomes after ART remains to be determined. Therefore, this population-based retrospective cohort study aimed to assess whether and how higher BMI affects singleton pregnancy outcomes.

**Methods:**

This study used the large nationally representative database of the US National Inpatient Sample (NIS), extracting data of women with singleton pregnancies who had received ART from 2005 to 2018. Diagnostic codes of the International Classification of Diseases, Ninth and Tenth edition (ICD-9 and ICD-10) were used to identify females admitted to US hospitals with delivery-related discharge diagnoses or procedures and secondary diagnostic codes for ART, including in vitro fertilization. The included women were further categorized into three groups based on BMI values < 30, 30–39, and ≥ 40 kg/m^2^. Univariate and multivariable regression analysis were conducted to assess the associations between study variables and maternal and fetal outcomes.

**Results:**

Data of totally 17,048 women were included in the analysis, which represented a population of 84,851 women in the US. Number of women in the three BMI groups were 15, 878 (BMI < 30 kg/m^2^), 653 (BMI 30–39 kg/m^2^), and 517 (BMI ≥ 40 kg/m^2^), respectively. The multivariable regression analysis revealed that, compared to BMI < 30 kg/m^2^, BMI 30–39 kg/m^2^ was significantly associated with increased odds for pre-eclampsia and eclampsia (adjusted OR = 1.76, 95% CI = 1.35, 2.29), gestational diabetes (adjusted OR = 2.25, 95% CI = 1.70, 2.98), and Cesarean delivery (adjusted OR = 1.36, 95% CI = 1.15, 1.60). Further, BMI ≥ 40 kg/m^2^ was associated with greater odds for pre-eclampsia and eclampsia (adjusted OR = 2.25, 95% CI = 1.73, 2.94), gestational diabetes (adjusted OR = 3.64, 95% CI = 2.80, 4.72), disseminated intravascular coagulation (DIC) (adjusted OR = 3.79, 95% CI = 1.47, 9.78), Cesarean delivery (adjusted OR = 1.85, 95% CI = 1.54, 2.23), and hospital stay ≥ 6 days (adjusted OR = 1.60, 95% CI = 1.19, 2.14). However, higher BMI was not significantly associated with greater risk of the fetal outcomes assessed.

**Conclusions:**

Among US pregnant women who received ART, having a higher BMI level independently increases the risk for adverse maternal outcomes such as pre-eclampsia and eclampsia, gestational diabetes, DIC, longer hospital stays, and higher rates of Cesarean delivery, while risk is not increased for fetal outcomes.

**Supplementary Information:**

The online version contains supplementary material available at 10.1186/s12884-023-05620-7.

## Background

Since 1980, the global prevalence of overweight and obesity has more than doubled, with nearly a third of the world’s population now classified as overweight or obese. Obesity rates have increased across all ages and genders, regardless of geographic location, ethnicity, or socioeconomic status, though the prevalence of obesity is generally higher in older people and women [1]. Obesity is a major public health issue because it significantly increases the risk of diseases such as type 2 diabetes, fatty liver disease, hypertension, myocardial infarction, stroke, dementia, osteoarthritis, obstructive sleep apnea, and several cancers, all of which contribute to a decline in both quality of life and life expectancy [2, 3].

Besides, it is widely known that obesity also impacts women’s reproductive health and pregnancy outcomes [4–9]. Women with obesity have a disruption in the hypothalamic pituitary ovarian axis, resulting in menstrual dysfunction, anovulation, and infertility [4–6]. Women with pre-pregnancy BMIs more than 25 kg/m^2^ are more likely to experience infertility issues and are at higher risk of miscarriage and stillbirth than those with ideal pre-pregnancy BMIs [9]. Overweight, obesity, and excessive gestational weight gain increase the likelihood of all pregnancy problems, including those that pose a serious risk to the lives of mothers and newborns [9].

Furthermore, previous studies reported that obesity also influences fertility treatment, specifically, the outcomes of assisted reproduction technology (ART) in women [10–14]. It was pointed out that increased BMI may reduce the likelihood of conception in ovulatory women and influences the outcome of ovulation induction treatment [10–12]. Also, women with obesity undergoing in vitro fertilization (IVF) require higher gonadotrophin doses, have poor ovarian stimulation response, and have fewer oocytes harvested [10–12]. Nevertheless, the researchers indicated that, to date, there is still no universal consensus on the detrimental effects of obesity on the female reproductive potential in ART cycles, and the pathophysiology underlying the effects deserves further investigation [13, 14].

Moreover, recent studies suggested that women with obesity who had ART for a singleton pregnancy were more likely to have a cesarean section, gestational diabetes mellitus, gestational hypertension, and preeclampsia [15, 16]. However, the evidence on the clinical impact of high BMI on various maternal and child-health outcomes in women receiving ART is not sufficient and remains to be clarified, particularly in a nationwide cohort. Given the rapidly expanded use of ART in women [17] and the healthcare burden that high BMI brings about to women at reproductive age, this study aimed to assess whether and how higher BMI affects singleton pregnancy outcomes in women undergoing ART, including maternal and child-helath outcomes, using a large nationally representative database from the United States (US) across 14 years.

## Methods

### Study design and data source

This study used data from the US Nationwide Inpatient Sample (NIS) database, which is a large all-payer, continuous inpatient care database in the United States that includes about 8 million hospital stays annually. The NIS is administered by the Healthcare Cost and Utilization Project (HCUP) of the US National Institutes of Health (NIH). The data includes patient demographics, primary and secondary diagnoses, primary and secondary procedures, expected payment source, duration of hospital stay, admission and discharge status, and hospital characteristics. The sample includes 1,050 hospitals from 44 states, representing a 20% stratified sample of US community hospitals as defined by the American Hospital Association. More information about the data set can be found on the NIS website: https://www.hcup-us.ahrq.gov/nisoverview.jsp.

### Ethics statement

All data were obtained through request to the Online Healthcare Cost and Utilization Project (HCUP) Central Distributor, which administers the database (certificate#HCUP-71HZP39M9). This study conforms to the NIS data-use agreement with HCUP. Because this study analyzed secondary data from the NIS database, patients and the public were not involved directly. Since all data in the NIS database are de-identified, the requirement for informed consent was also waived. The study protocol was also reviewed and the ethical approval was waived by the Shin Kong Wu Ho-Su Memorial Hospital (SKH) Institutional Review Board.

### Selection of study population

The International Classification of Diseases, Ninth and Tenth edition (ICD-9 and ICD-10) diagnostic codes were used to identify females admitted to US hospitals from 2005 to 2018 with delivery-related discharge diagnoses or procedures, and secondary diagnostic codes of ART, including in vitro fertilization (IVF) and intracytoplasmic sperm injection (ICSI). Women with multiple pregnancies were excluded. Included women were further categorized into three groups according to BMI categories: <30, 30–39, and ≥ 40 kg/m^2^, which indicated non-obese, obese and severe obese, respectively, based on the definition provided by the Centers for Diseases Control and Prevention of the US (https://www.cdc.gov/obesity/basics/adult-defining.html).

### Study variables and outcome measures

Two outcome categories were assessed: maternal and fetal. Maternal outcomes included pre-eclampsia and eclampsia, gestational diabetes, antepartum hemorrhage, placenta previa, preterm premature rupture of membrane (PPROM), chorioamnionitis (CAM), forceps, Cesarean delivery, post-partum hemorrhage, disseminated intravascular coagulation (DIC), venous thromboembolism (VTE), post-partum hysterectomy, transfusion, and hospital stays ≥ 6 days. Fetal outcomes included stillbirth and intrauterine fetal death (IUFD), intrauterine growth restriction (IUGR), premature birth, large-for-gestational age (LGA), birth defects, and abortion. The detailed ICD codes for identifying the said conditions are summarized in Supplementary Table S1.

### Covariates

Patients’ characteristics included age (< 25; 25–35; >35 years), race, and insurance status (primary payer). Smoking status (ICD-9: 305.1, V15.82, 989.84; ICD-10: Z71.6, Z72.0, Z86.43, Z87.891, F17, O99.33, T65.2), PCOS (ICD9: 256.4; ICD10: E28.2), and major comorbidities were identified using the same ICD coding system. Comorbidities included in this analysis were: coronary artery disease, congestive heart failure, pre-existing diabetes, hypertension, cerebrovascular disease, chronic pulmonary disease, rheumatic disease, chronic kidney disease, coagulopathy and thyroid disorder. Hospital-related characteristics such as bed size, location/teaching status, and hospital region were extracted from the database as part of the comprehensive data available for all participants.

### Statistical analysis

Descriptive statistics of the study cohort are presented as numbers (n) and weighted percentages (%) for categorical data and mean and standard error (SE) for continuous data. Logistic regression analyses were performed to determine the odds ratios (OR) and 95% confidence intervals (CI) of maternal BMI on study outcomes. The multivariable model was adjusted for age, race, income, primary payer, smoking, PCOS, number of comorbidities, hospital bed size, hospital location/teaching status, and hospital region. Since the NIS database covers 20% samples of the US annual inpatient admissions, weighted samples (before 2011 using Trend WT & after 2012 using DISCWT), stratum (NIS_STRATUM), cluster (HOSPID) were used to produce national estimates for all analyses. All p values were two-sided and p < 0.05 was considered statistically significant. All statistical analyses were performed using the statistical software package SAS software version 9.4 (SAS Institute Inc., Cary, NC, USA).

## Results

### Study population

**Fig. 1:**
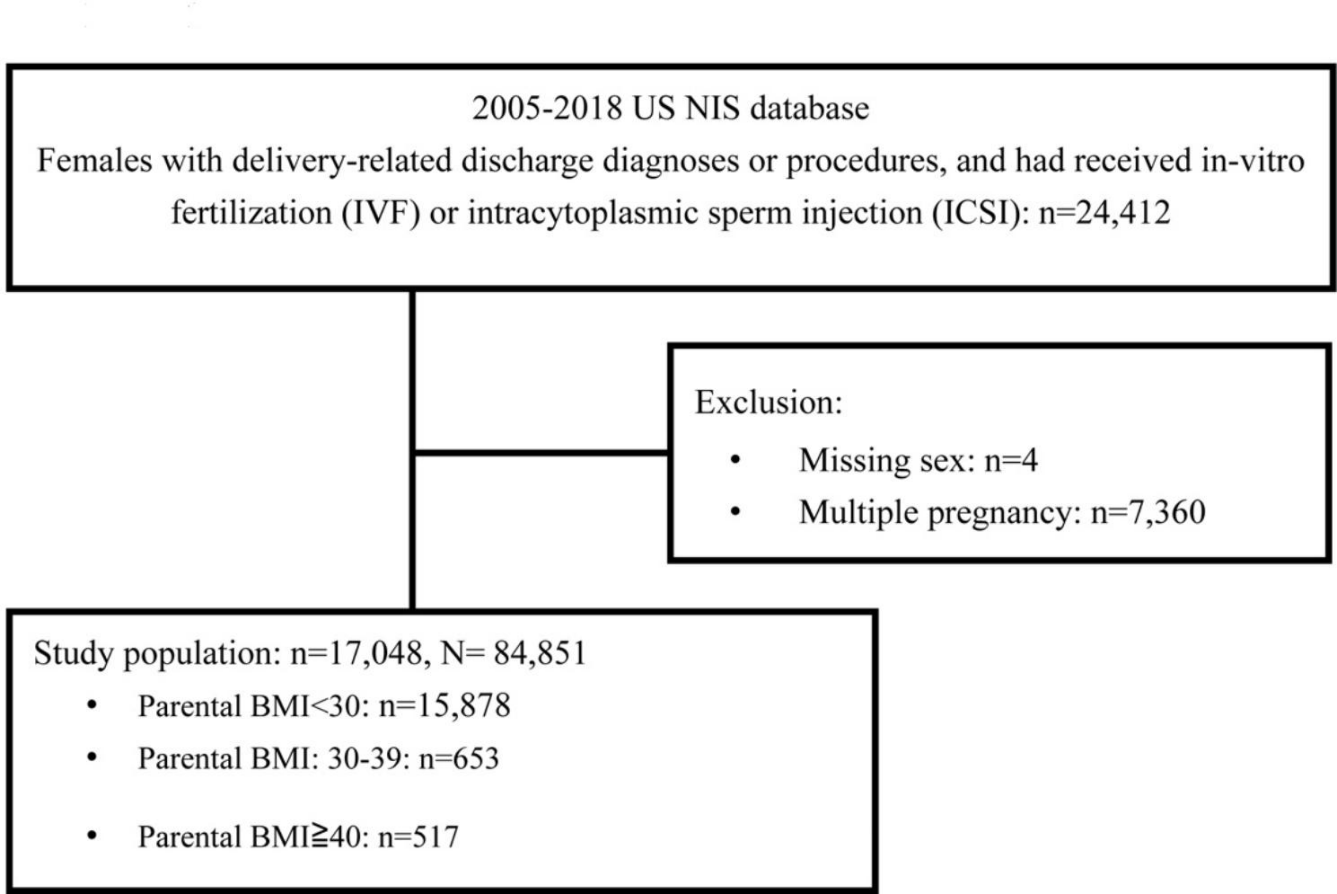
Flow chart of the study population

The data of 24,412 pregnant females listed in the NIS database as receiving ART between 2005 and 2018 were initially included. Individuals with missing sex (n = 4) or having a multiple pregnancy (n = 7,360) were excluded. Finally, 17,048 women were enrolled as the primary cohort whose data were included in the analysis, representing a population of 84,851 women in the US (Fig. 1).

### Characteristics of the study cohort

Demographics, comorbidities, and hospital characteristics of the study cohort are summarized in Table 1. Among the study population, number of women in the three groups were 15, 878 (BMI < 30), 653 (BMI 30–39), and 517 (BMI ≥ 40). Half of the women were older than 35 years. Most were Whites (69.4%), with higher household income (55.7%), with insurance covered by private primary payer (91.9%), without comorbidities (73.0%), staying in large bed-size hospitals (57.8%) and urban-teaching hospitals (74.6%). The proportions of women with PCOS in the three groups were 3.4% (BMI < 30), 10.5% (BMI 30–39) and 14.3% (BMI ≥ 40), respectively (p < 0.001). The most prevalent comorbidity was thyroid disorder (16.9%), which were differently distributed in the three groups: 16.7% (BMI < 30), 18.2% (BMI 30–39) and 22.8% (BMI ≥ 40) (p < 0.001). (Table 1)

**Table 1:** Baseline demographic and clinical characteristics of mothers by BMI category

Characteristics	Total (n = 17,048)	Maternal BMI, kg/m^2^			P-value
		< 30 (n = 15,878)	30–39 (n = 653)	≥ 40 (n = 517)	
**Demography**					
Age					
< 25	179 (1.0)	161 (1.0)	10 (1.6)	8 (1.6)	0.166
25–35	8300 (48.7)	7703 (48.6)	328 (50.2)	269 (51.9)	
> 35	8569 (50.2)	8014 (50.4)	315 (48.3)	240 (46.5)	
Race					
White	11,129 (69.4)	10,384 (69.5)	395 (65.8)	350 (70.9)	**< 0.001**
Black	1015 (6.3)	897 (6.0)	57 (9.4)	61 (12.3)	
Hispanic	1064 (6.6)	960 (6.4)	69 (11.5)	35 (7.0)	
Others	2835 (17.6)	2708 (18.1)	79 (13.2)	48 (9.7)	
Missing	1005	929	53	23	
Household income					
Quartile1	1237 (7.3)	1125 (7.2)	63 (9.7)	49 (9.5)	**< 0.001**
Quartile2	2138 (12.7)	1949 (12.4)	112 (17.4)	77 (15.1)	
Quartile3	4111 (24.3)	3775 (24.0)	182 (28.1)	154 (30.1)	
Quartile4	9416 (55.7)	8891 (56.5)	291 (44.9)	234 (45.3)	
Missing	146	138	5	3	
Primary Payer					
Medicare/Medicaid	767 (4.5)	699 (4.4)	36 (5.5)	32 (6.2)	0.153
Private including HMO	15,661 (91.9)	14,594 (91.9)	595 (91.1)	472 (91.3)	
Self-pay/no-charge/other	609 (3.6)	574 (3.6)	22 (3.4)	13 (2.5)	
Missing	11	11	0	0	
Smoking	642 (3.8)	548 (3.5)	57 (8.7)	37 (7.1)	**< 0.001**
PCOS	682 (4.0)	539 (3.4)	69 (10.5)	74 (14.3)	**< 0.001**
**Number of comorbidities**					
0	12,449 (73.0)	11,725 (73.8)	433 (66.3)	291 (56.3)	**< 0.001**
1 ~ 2	4549 (26.7)	4114 (25.9)	216 (33.1)	219 (42.3)	
3+	50 (0.3)	39 (0.2)	4 (0.6)	7 (1.4)	
**Comorbidities**					
Coronary artery disease	3 (0.0)	2 (0.0)	1 (0.2)	0 (0.0)	-
Congestive heart failure	8 (0.0)	6 (0.0)	1 (0.2)	1 (0.2)	0.117
Diabetes	211 (1.2)	158 (1.0)	20 (3.0)	33 (6.4)	**< 0.001**
Hypertension	440 (2.6)	335 (2.1)	45 (6.9)	60 (11.6)	**< 0.001**
Cerebrovascular disease	12 (0.1)	12 (0.1)	0 (0.0)	0 (0.0)	-
Chronic pulmonary disease	859 (5.0)	751 (4.7)	54 (8.3)	54 (10.4)	**< 0.001**
Rheumatic disease	94 (0.6)	89 (0.6)	3 (0.5)	2 (0.4)	0.844
Chronic kidney disease	16 (0.1)	15 (0.1)	0 (0.0)	1 (0.2)	-
Coagulopathy	690 (4.1)	657 (4.1)	22 (3.3)	11 (2.1)	**0.047**
Thyroid disorder	2881 (16.9)	2645 (16.7)	118 (18.2)	118 (22.8)	**< 0.001**
**Hospital information**					
Hospital bed size					
Small	2340 (13.4)	2184 (13.4)	84 (12.8)	72 (13.9)	0.656
Medium	4877 (28.8)	4556 (28.9)	171 (26.3)	150 (29.0)	
Large	9816 (57.8)	9124 (57.6)	398 (61.0)	294 (57.1)	
Missing	15	14	0	1	
Hospital location/teaching status					
Rural	462 (2.7)	435 (2.7)	12 (1.8)	15 (2.9)	**0.030**
Urban nonteaching	3882 (22.7)	3654 (22.9)	126 (19.2)	102 (19.7)	
Urban teaching	12,689 (74.6)	11,775 (74.4)	515 (79.0)	399 (77.4)	
Missing	15	14	0	1	
Hospital region					
Northeast	5844 (34.5)	5465 (34.6)	206 (31.6)	173 (33.6)	0.095
Midwest	2566 (15.0)	2353 (14.8)	118 (18.0)	95 (18.3)	
South	4171 (24.3)	3898 (24.4)	145 (22.1)	128 (24.6)	
West	4467 (26.2)	4162 (26.2)	184 (28.2)	121 (23.5)	

BMI, body mass index; PCOS, polycystic ovary syndrome

Categorical variables are presented as unweighted counts (weighted percentage)

Continuous data are presented as mean ± SE.

P-values < 0.05 are shown in bold

Maternal outcomes and fetal outcomes are shown in Table 2. The proportions of women who had pre-eclampsia and eclampsia, gestational diabetes, received Cesarean delivery, DCI, and stayed in hospitals ≥ 6 days were significantly larger in the higher BMI groups (BMI 30–39 and ≥ 40 kg/m^2^) than in the BMI < 30 group. No significantly different distributions regarding fetal outcomes were observed between different BMI groups. (Table 2)

**Table 2:** Outcomes of mothers by BMI category

Characteristics	Total (n = 17,048)	Maternal BMI, kg/m^2^			P-value
		< 30 (n = 15,878)	30–39 (n = 653)	≥ 40 (n = 517)	
**Maternal outcomes**					
**Antepartum period**					
Pre-eclampsia and eclampsia	1210 (7.1)	1049 (6.6)	79 (12.1)	82 (15.9)	**< 0.001**
Gestational diabetes	850 (5.0)	695 (4.4)	71 (10.8)	84 (16.3)	**< 0.001**
Antepartum hemorrhage	141 (0.8)	138 (0.9)	0 (0.0)	3 (0.6)	-
Placenta previa	885 (5.2)	833 (5.3)	36 (5.5)	16 (3.2)	0.122
PPROM	1444 (8.5)	1347 (8.5)	52 (7.9)	45 (8.7)	0.861
CAM	201 (1.2)	184 (1.2)	12 (1.8)	5 (1.0)	0.259
**Intrapartum period**					
Forceps	133 (0.8)	125 (0.8)	4 (0.6)	4 (0.8)	0.858
Cesarean delivery	8001 (46.9)	7335 (46.2)	349 (53.5)	317 (61.4)	**< 0.001**
**Post-partum period**					
Post-partum hemorrhage	1059 (6.2)	979 (6.2)	48 (7.4)	32 (6.1)	0.467
DIC	61 (0.4)	54 (0.3)	1 (0.2)	6 (1.2)	**0.009**
VTE	203 (1.2)	189 (1.2)	6 (0.9)	8 (1.5)	0.629
Post-partum hysterectomy	3 (0.0)	3 (0.0)	0 (0.0)	0 (0.0)	-
Transfusion	1 (0.0)	1 (0.0)	0 (0.0)	0 (0.0)	-
Hospital stay ≥ 6 days	1208 (7.1)	1085 (6.9)	60 (9.2)	63 (12.2)	**< 0.001**
**Fetal outcomes**					
Stillbirth and IUFD	138 (0.8)	124 (0.8)	6 (0.9)	8 (1.6)	0.095
IUGR	661 (3.9)	615 (3.9)	26 (4.0)	20 (3.9)	0.988
Premature birth	1359 (8.0)	1271 (8.0)	51 (7.8)	37 (7.2)	0.791
LGA	0 (0.0)	0 (0.0)	0 (0.0)	0 (0.0)	-
Birth defect	300 (1.8)	280 (1.8)	13 (2.0)	7 (1.4)	0.754
Abortion	118 (0.7)	105 (0.7)	9 (1.4)	4 (0.8)	0.085

BMI, body mass index; PPROM, preterm premature rupture of membranes; CAM, Chorioamnionitis; DIC, disseminated intravascular coagulation; VTE, venous thromboembolism; IUFD, intrauterine fetal death; IUGR, intrauterine growth restriction; LGA, Large-for-gestational age; PCOS, polycystic ovary syndrome

Categorical variables are presented as unweighted counts (weighted percentage)

Continuous data are presented as mean ± SE.

P-values < 0.05 are shown in bold

### Associations between BMI and maternal outcomes (BMI ≥ 40 and 30–39 versus < 30 kg/m^2^)

The relationships between maternal BMI categories and maternal outcomes of pregnancy after ART are listed in Table 3. After adjusting for covariates, women with BMI between 30 and 39 kg/m^2^ were significantly more likely to have pre-eclampsia and eclampsia (adjusted OR = 1.76, 95% CI = 1.35, 2.29), gestational diabetes (adjusted OR = 2.25, 95% CI = 1.70, 2.98) during pregnancy, and were more likely to have Cesarean delivery (adjusted OR = 1.36, 95% CI = 1.15, 1.60). Similarly, women with a BMI ≥ 40 kg/m^2^ had greater odds for developing pre-eclampsia and eclampsia (adjusted OR = 2.25, 95% CI = 1.73, 2.94), gestational diabetes (adjusted OR = 3.64, 95% CI = 2.80, 4.72), DIC (adjusted OR = 3.79, 95% CI = 1.47, 9.78), receiving Cesarean delivery (adjusted OR = 1.85, 95% CI = 1.54, 2.23), and with hospital stays ≥ 6 days (adjusted OR = 1.60, 95% CI = 1.19, 2.14). (Table 3)

**Table 3:** Associations between BMI and maternal outcomes

Variables	Univariate OR (95% CI)		Adjusted OR ^a^ (95% CI)	
	BMI 30–39 kg/m^2^ (vs. < 30 kg/m^2^)	BMI ≥ 40 kg/m^2^ (vs. < 30 kg/m^2^)	BMI 30–39 kg/m^2^ (vs. < 30 kg/m^2^)	BMI ≥ 40 kg/m^2^ (vs. < 30 kg/m^2^)
**Maternal outcomes**				
Antepartum period				
Pre-eclampsia and eclampsia	**1.94 (1.51, 2.48)**	**2.67 (2.09, 3.42)**	**1.76 (1.35, 2.29)**	**2.25 (1.73, 2.94)**
Gestational diabetes	**2.62 (2.02, 3.40)**	**4.23 (3.34, 5.36)**	**2.25 (1.70, 2.98)**	**3.64 (2.80, 4.72)**
Placenta previa	1.06 (0.75, 1.50)	0.59 (0.35, 1.00)	0.93 (0.64, 1.36)	0.62 (0.36, 1.07)
PPROM	0.93 (0.70, 1.23)	1.02 (0.75, 1.38)	0.92 (0.68, 1.24)	1.06 (0.77, 1.46)
CAM	1.59 (0.89, 2.84)	0.83 (0.34, 2.02)	1.40 (0.76, 2.58)	0.81 (0.33, 1.98)
Intrapartum period				
Forceps	0.77 (0.32, 1.84)	0.99 (0.37, 2.68)	0.88 (0.37, 2.10)	1.12 (0.41, 3.11)
Cesarean delivery	**1.34 (1.15, 1.57)**	**1.85 (1.55, 2.21)**	**1.36 (1.15, 1.60)**	**1.85 (1.54, 2.23)**
Post-partum period				
Post-partum hemorrhage	1.21 (0.89, 1.63)	0.99 (0.69, 1.43)	1.14 (0.84, 1.56)	0.99 (0.68, 1.45)
DIC	0.45 (0.06, 3.22)	**3.41 (1.37, 8.52)**	0.47 (0.06, 3.51)	**3.79 (1.47, 9.78)**
VTE	0.78 (0.35, 1.74)	1.30 (0.64, 2.63)	0.79 (0.35, 1.77)	1.20 (0.58, 2.47)
Hospital stay ≥ 6 days	**1.38 (1.05, 1.80)**	**1.89 (1.44, 2.50)**	1.19 (0.89, 1.59)	**1.60 (1.19, 2.14)**

BMI, body mass index; CI, confidence interval; OR, odds ratio; PPROM, preterm premature rupture of membranes; CAM, Chorioamnionitis; DIC, disseminated intravascular coagulation; VTE, venous thromboembolism

P-values < 0.05 are shown in bold

^a^ Adjusted for age, race, household income, primary payer, smoking, PCOS, number of comorbidities, hospital bed size, hospital location/teaching status, and hospital region

### Associations between BMI and fetal outcomes (BMI ≥ 40 and 30–39 versus < 30 kg/m^2^)

The relationships between maternal BMI categories and maternal outcomes of pregnancy following ART are listed in Table 4. After adjusting for relevant confounders, the results revealed that higher BMI was not significantly associated with greater risks for the fetal outcomes assessed. (Table 4)

**Table 4:** Associations between BMI and fetal outcomes

Variables	Univariate OR (95% CI)		Adjusted OR ^a^ (95% CI)	
	BMI 30–39 kg/m^2^ (vs. < 30 kg/m^2^)	BMI 40 + kg/m^2^ (vs. < 30 kg/m^2^)	BMI 30–39 kg/m^2^ (vs. < 30 kg/m^2^)	BMI 40 + kg/m^2^ (vs. < 30 kg/m^2^)
**Fetal outcomes**				
Stillbirth and IUFD	1.18 (0.59, 2.34)	2.02 (0.99, 4.12)	1.12 (0.56, 2.26)	1.83 (0.88, 3.80)
IUGR	1.03 (0.70, 1.52)	1.00 (0.64, 1.58)	1.02 (0.68, 1.53)	0.96 (0.61, 1.52)
Premature birth	0.97 (0.72, 1.30)	0.89 (0.64, 1.24)	0.89 (0.64, 1.23)	0.86 (0.62, 1.21)
Birth defect	1.12 (0.65, 1.96)	0.79 (0.37, 1.69)	1.18 (0.68, 2.07)	0.63 (0.28, 1.41)
Abortion	**2.10 (1.10, 4.01)**	1.18 (0.43, 3.21)	1.88 (0.93, 3.78)	1.13 (0.40, 3.17)

BMI, body mass index; CI, confidence interval; OR, odds ratio; IUFD, intrauterine fetal death; IUGR, intrauterine growth restriction

P-values < 0.05 are shown in bold

^a^ Adjusted for age, race, household income, primary payer, smoking, PCOS, number of comorbidities, hospital bed size, hospital location/teaching status, and hospital region

## Discussion

This study assessed whether higher BMI affects the outcomes of singleton pregnancy in women receiving ART, using a large 14-year nationally representative database in the US. Maternal and fetal outcomes were compared between women with BMI ≥ 40 and 30–39 to < 30 kg/m^2^. As a result, we found that higher BMI category posed a greater risk for adverse maternal outcomes. After adjusting for relevant confounders, compared to BMI < 30 kg/m^2^, BMI 30–39 kg/m^2^ is independently associated with a 1.76-fold greater risk for pre-eclampsia and eclampsia, 2.25-fold risk for gestational diabetes, and 1.36-fold risk for Cesarean delivery. Furthermore, BMI ≥ 40 kg/m^2^ is independently associated with a 2.25-fold greater risk for pre-eclampsia and eclampsia, 3.64-fold for gestational diabetes, 1.85-fold for Cesarean delivery, 3.79-fold for DIC, and 1.60-fold for prolonged hospital stay ≥ 6 days than BMI < 30 kg/m^2^. However, after adjusting for relevant confounders, higher maternal BMI category was not significantly associated with greater risks for the fetal outcomes assessed (i.e., stillbirth, IUFD, IUGR, premature birth, LGA, birth defects and abortion) than BMI < 30 kg/m^2^. The results indicate that compared to non-obese, high BMI meeting the definitions of either obese or severe obese, poses greater risks for several adverse maternal pregnancy outcomes among women receiving ART, and severe obese has even greater impact.

The prevalence of obesity, defined by the World Health Organization (WHO) as a BMI ≥ 30 kg/m^2^, is a global epidemic and has become a serious health problem [1–3]. For women’s reproductive health, obesity is also an independent risk factor for infertility [4–9]. Moreover, although ART has become an integral part of modern medicine, the lower success rates and higher miscarriage rates after ART in women with obesity than women with normal BMI were continuously reported [10–14].

Most of the previous studies focused on the impact of obesity on pregnancy rates or miscarriage rates in women receiving ART, instead of maternal-related outcomes or child-health outcomes as assessed in the present study. For instance, a previous systematic review included 49 studies and concluded thatwomen with obesity (BMI ≥ 30 kg/m^2^) have a significant higher miscarriage rate and lower live birth rate following ART when compared to women with a normal BMI [13]. Another recent meta-analysis also concluded that being overweight or obese has a weak adverse impact on clinical pregnancy rates, live birth and miscarriage rates, number of mature oocytes, duration of ovarian stimulation, as well as gonadotropin dosage used [14], indicating losing weight before ART treatment might be of clinical benefits.

On the other hand, in a total of 18,687,217 delivery-related hospitalizations during 2010–2014 among general pregnant population in the US NIS database, the prevalence of cesarean delivery and gestational diabetes in women with obesity were 52.8% and 15.8% [18]. Similarly, in the present analysis limited to women receiving ART, the prevalence of cesarean delivery and gestational diabetes and in women with a BMI > 30 were 57% and 13%. When considering the impact of obesity after adjustment, in the general population, women with obesity were more likely to have cesarean deliveries (aOR 1.70) and labor inductions (aOR 1.51), greater length of stay after cesarean deliveries (aOR 1.14) and vaginal deliveries (aOR 1.48), pregnancy-related hypertension (aOR 2.17), preeclampsia (aOR 2.06), gestational diabetes (aOR 2.75), PPROM (aOR 1.17), chorioamnionitis (aOR 1.39), and venous thromboembolism (aOR 1.63), but not fetal chromosomal abnormalities or stillbirth [18]. In our analysis, in women receiving ART, the risks that obesity (BMI 30–39) posed on cesarean delivery, pre-eclampsia and eclampsia, and gestational diabetes falls between 1.3 and 2.4, which seemed quite similar to the general population.

A recent study by Sun et al. included 3,043 Chinese women across 2015 to 2020. The women were subdivided into underweight (BMI < 18.5 kg/m^2^), normal (BMI 18.5 to < 23 kg/m^2^), overweight (BMI 23 to < 27.5 kg/m^2^), and obese (BMI ≥ 27.5 kg/m^2^) according to the Asian criteria of obesity. The authors concluded that women with overweight or obesity who had ART for a singleton pregnancy were significantly more likely to have a cesarean section, gestational diabetes mellitus, gestational hypertension, and preeclampsia. Furthermore, neonates born to mothers with obesity were more likely to have macrosomia [15]. Despite querying different population, the findings of Sun et al. are generally in line with those of the present analysis. However, while Sun et al. did not analyze the impact of severe obesity, probably due to not as prevalent in Chinese women as in American women, the present analysis found that severe obesity (BMI ≥ 40 kg/m^2^) is associated with even greater odds for those adverse maternal outcomes, providing a clearer picture on the dose-dependent effect that increasing BMI might bring about.

Another study by Qu et al. included 7,818 women undergoing ART and their singleton infants in one single ART center. It was reported that in ART-conceived singletons, pre-pregnancy maternal overweight and obesity were associated with increased risks of preterm birth, macrosomia, and LGA. The authors also indicated that the timing of embryo transfer had an effect on these associations and suggested women to maintain a normal BMI prior to ART in order to avoid adverse perinatal outcomes [16]. Inconsistently, our analysis did not observe significant associations between high maternal BMI and adverse fetal outcomes including stillbirth, IUFD, IUGR or LGA. A possible explain is that the NIS database is an inpatient data set, and such fetal outcomes were not necessarily assessed at the hospital setting thus were not fully captured. Future studies using data from a specific ART registry to repetitively assess these outcomes are recommended.

Interestingly, recent studies reported that obesity was linked to poorer IVF outcomes, namely, decreased rates of pregnancy and live birth, in women who had their first frozen-thawed embryo transfer (FET) [19], as well as greater risk for pre-eclampsia [20, 21]. Given there is no detail information to distinguish whether a fresh embryo transfer or a FET was applied in the NIS database, we were not able to analyze the potential impact of higher BMI on maternal outcomes after a FET. Future studies are highly warranted to address this issue.

### Strengths and limitations

The present study is inherently limited by its retrospective and observational nature. The possibility of coding errors has been noted in other studies that used the ICD coding system. Also, BMI could not be further subdivided, e.g., into 30-34.9 and 35-39.9 based on the coding system used. Although it may be important, the causes of infertility and types of ART techniques could not be identified thus could not be adjusted for. This study also lacks data of clinical laboratory parameters and long-term follow-up data after discharge, which were not available in the NIS database. Despite these limitations, the demographic factors, comorbidities, and hospital-level variables were considered and carefully adjusted in this analysis, lending additional credibility to the findings. Also, this study provided evidence from a large nationally representative sample based on the NIS data, which the findings are likely generalizable to the whole population of the US.

## Conclusions

In US pregnant women who received ART, higher BMI significantly increases the risk for adverse maternal outcomes such as pre-eclampsia and eclampsia, gestational diabetes, DIC, longer hospital stays, and rates of Cesarean delivery. However, no significant associations between higher maternal BMI and fetal outcomes were observed.

## Electronic supplementary material

Below is the link to the electronic supplementary material.

Additional file 1: Supplementary Table S1

## Data Availability

All data analysed during this study are included in this published article.

## List of abbreviations

ART: Assisted reproductive technology
BMI: Body mass index
NIS: National Inpatient Sample
DIC: Disseminated intravascular coagulation
IVF: In vitro fertilization
HCUP: Healthcare Cost and Utilization Project
ICSI: Intracytoplasmic sperm injection
