# Supplementary material for: Impact of maternal body mass index on outcomes of singleton pregnancies after assisted reproductive technology: a 14-year analysis of the US Nationwide Inpatient Sample
Source: BMC Pregnancy Childbirth. 2023 Apr 26;23:291. doi: 10.1186/s12884-023-05620-7 (PMC10131305; doi:10.1186/s12884-023-05620-7)
Supplement: Supplementary file 1 — Additional file 1: Supplementary Table S1 [file 12884_2023_5620_MOESM1_ESM.docx]

Supplementary Table S1. Definitions and International Classification of Disease-9th Revision (ICD-9) codes and Disease-10th Revision (ICD-10) codes used.

|  | Definitions | ICD-9-CM / ICD-9-PCS | ICD-10-CM / ICD-10-PCS |
| --- | --- | --- | --- |
| Delivery-related discharge diagnoses or procedures or maternal deaths | Defined from diagnosis | 650-677, 634-679, 761-766 ICD-9-PCS: 72-75 | O30-O48, O60-O77, O80-O82, O82-O92,  O94-O9A, P00-P96, Z37, Z38 ICD-10-PCS: 1090, 10D, 10E0 |
| In vitro fertilization (IVF) / intracytoplasmic sperm injection (ICSI) | Defined from diagnosis | V23.85, V26.81 | N98, Z31.83, O09.81 ICD-10-PCS: 8E0ZXY1 |
| Multiple pregnancy | Defined from diagnosis | 651 | O30, O31, Z37.2 - Z37.7, Z38.3 - Z38.8 |
| BMI<30 (Overweight) | Defined from diagnosis | 278.02, V85.2 | E66.3, Z68.25-Z68.29 |
| BMI 30-39 (Obese) | Defined from diagnosis | 278.00, V85.3 | E66.02, E66.09, E66.1, E66.8, E66.9, Z68.3 |
| BMI 40+ (Morbid obese) | Defined from diagnosis | 278.01, 278.03, V85.4 | E66.01, E66.2, Z68.4 |
| Pre-eclampsia and eclampsia | Defined from diagnosis | 642.4-642.7 | O14, O15 |
| Gestational diabetes | Defined from diagnosis | 648 | O24 |
| Antepartum hemorrhage | Defined from diagnosis | 641.8-641.9 | O46 |
| Placenta previa | Defined from diagnosis | 641.0-641.3 | O44 |
| PPROM | Defined from diagnosis | 761.1, 658.1 | O42 |
| CAM | Defined from diagnosis | 762.7 | O41.12 |
| Forceps | Defined from diagnosis | 763.2, 660.71, 669.51 | O66.5, P03.2 |
| Cesarean delivery | Defined from diagnosis | 763.4, 669.7, V30.01 ICD-9-PCS: 74 | P03.4, O82, Z38.01 ICD-10-PCS: 10D00Z0-10D00Z3 |
| Post-partum hemorrhage | Defined from diagnosis | 666 | O72 |
| DIC | Defined from diagnosis | 286.6 | D65 |
| VTE | Defined from diagnosis | 415, 451, 452, 453, 671, 673 | I26, I80, I81, I82, O87, O88 |
| Post-partum hysterectomy | Defined from diagnosis | V88.0 | Z90.71 |
| Transfusion | Defined from diagnosis | 999.4-999.8, 276.61 ICD-9-PCS: 99 | T80,3, T80.4, T80.A, T80.5, T80.6, T80.91, T80.92 E87.71 ICD-10-PCS: 3023, 3024, 3027, 3028 |
| Still birth and IUFD | Defined from diagnosis | 656.4, V27.1 | O36.4, Z37.1, P95 |
| IUGR | Defined from diagnosis | 656.5, V28.4, 764 | O36.59, Z36.4, P05 |
| Premature birth | Defined from diagnosis | 644.2, 765 | O60, P07 |
| LGA (Large-for-gestational age) | Defined from diagnosis | 766 | P08 |
| Birth defect | Defined from diagnosis | 740-759 | Q00-Q99 |
| Abortion | Defined from diagnosis | 630-639 | O00-O08 |
| Smoking | Defined from diagnosis | 305.1, 989.84, V15.82 | Z71.6, Z72.0, Z86.43, Z87.891, F17, O99.33, T65.2 |
| PCOS | Defined from diagnosis | 256.4 | E28.2 |

BMI, body mass index; PPROM, preterm premature rupture of membranes; CAM, Chorioamnionitis; DIC, disseminated intravascular coagulation; VTE, venous thromboembolism; IUFD, intrauterine fetal death; IUGR, intrauterine growth restriction; LGA, Large-for-gestational age; PCOS, polycystic ovary syndrome.
